# N-Glycans Mediate the Ebola Virus-GP1 Shielding of Ligands to Immune Receptors and Immune Evasion

**DOI:** 10.3389/fcimb.2020.00048

**Published:** 2020-03-06

**Authors:** Muhammed Iraqi, Avishay Edri, Yariv Greenshpan, Kiran Kundu, Priyanka Bolel, Avishag Cahana, Aner Ottolenghi, Roi Gazit, Leslie Lobel, Alex Braiman, Angel Porgador

**Affiliations:** ^1^The Shraga Segal Department of Microbiology, Immunology and Genetics, Faculty of Health Sciences, Ben-Gurion University of the Negev, Be'er Sheva, Israel; ^2^National Institute for Biotechnology in the Negev, Ben-Gurion University of the Negev, Be'er Sheva, Israel

**Keywords:** Ab—antibody, EBOV—ebola virus, GP—glycoprotein, N-glycosylation, immune evasion, steric shielding

## Abstract

The Ebola Virus (EBOV) glycoprotein (GP) sterically shields cell-membrane ligands to immune receptors such as human leukocyte antigen class-1 (HLA-I) and MHC class I polypeptide-related sequence A (MICA), thus mediating immunity evasion. It was suggested that the abundant N-glycosylation of the EBOV-GP is involved in this steric shielding. We aimed to characterize (i) the GP N-glycosylation sites contributing to the shielding, and (ii) the effect of mutating these sites on immune subversion by the EBOV-GP. The two highly glycosylated domains of GP are the mucin-like domain (MLD) and the glycan cap domain (GCD) with three and six N-glycosylation sites, respectively. We mutated the N-glycosylation sites either in MLD or in GCD or in both domains. We showed that the glycosylation sites in both the MLD and GCD domains contribute to the steric shielding. This was shown for the steric shielding of either HLA-I or MICA. We then employed the fluorescence resonance energy transfer (FRET) method to measure the effect of N-glycosylation site removal on the distance in the cell membrane between the EBOV-GP and HLA-I (HLA.A^*^0201 allele). We recorded high FRET values for the interaction of CFP-fused HLA.A^*^0201 and YFP-fused EBOV-GP, demonstrating the very close distance (<10 nm) between these two proteins on the cell membrane of GP-expressing cells. The co-localization of HLA-I and Ebola GP was unaffected by the disruption of steric shielding, as the removal of N-glycosylation sites on Ebola GP revealed similar FRET values with HLA-I. However, these mutations directed to N-glycosylation sites had restored immune cell function otherwise impaired due to steric shielding over immune cell ligands by WT Ebola GP. Overall, we showed that the GP-mediated steric shielding aimed to impair immune function is facilitated by the N-glycans protruding from its MLD and GCD domains, but these N-glycans are not controlling the close distance between GP and its shielded proteins.

## Introduction

The Zaire Ebola Virus (EBOV) is one of the five known viruses within the EBOV genus (Kuhn et al., 2010). In many cases, EBOV, along with three other EBOVs, causes a severe pathology in humans and other mammals, which manifests as a hemogenic fever known as Ebola Virus Disease (EVD). EBOV is the sole cause of practically all human deaths from EVD. It recently erupted in the EBOV epidemic that struck West Africa in the years 2014–2016 (Na et al., 2015), resulting in at least 28,000 suspected cases and ~11,000 confirmed deaths in only 2 years (Leroy et al., 2009; Lori et al., 2015). A currently ongoing outbreak was reported in August 2018 in the Democratic Republic of Congo. EBOV has a negative-sense single-stranded RNA genome contained in filament-like viral particles composed of a viral envelope, matrix, and nucleocapsid components. Particles are ~80 nm in diameter, and the viral envelope carries virally encoded glycoproteins (GPs) (Brinkmann et al., 2016) projecting as 7- to 10-nm-long spikes from their lipid bilayer surfaces.

GP is produced in virally infected or artificially transfected cells thru a somewhat elaborate process. At first, an N-glycosylated precursor form of GP (GPpre) is translated; it is then transferred to the Golgi apparatus where it becomes fully glycosylated precursor GP. Hereafter, it is then cleaved by the convertase furin into two subunits: a ~120-kDa highly glycosylated fragment designated GP1 and a ~24-kDa fragment designated GP2; these are linked by disulfide bonds to produce the heterodimer form of membrane associated GP, which further goes through trimerization before appearing on the membrane (Sanchez et al., 1993; Volchkov et al., 1998). Sudan EBOV GP1 contains three domains, the receptor binding domain (RBD), which contains only one N-glycosylation site, the mucin-like domain (MLD), which contains three N-glycosylation sites, and the glycan cap domain (GCD), which contains six N-glycosylation sites (Sanchez et al., 2004).

The Ebola GP is known to sterically block or shield a variety of surface proteins in GP-expressing cells as shown in both virally infected and GP-transfected cells. The onset of steric shielding over immunologically significant molecules, *in vitro*, typically occurs between 24 and 48 h post infection along with maximal release of viral particles and viral protein secretion (VP24 and sGP). The phenotype of virally infected cells shows great resemblance to that of transiently transfected cells (Alazard-Dany et al., 2006). Among the molecules known to be shielded and blocked by Ebola GP are the human leukocyte antigen class-1 (HLA-I) molecules (Francica et al., 2010; Noyori et al., 2013). Peptide-presenting HLA-I are the ligands for the T cell receptor but HLA-I also serve as ligands for NK inhibitory receptors (Kärre et al., 1986; Ljunggren and Kärre, 1990; Colonna and Samaridis, 1995; D'andrea et al., 1995; Wagtmann et al., 1995; Braud et al., 1998; Lanier, 1998; Davis et al., 1999; López-Botet and Bellón, 1999; Kim et al., 2005; Vivier et al., 2011). We previously investigated the effect of EBOV-GP on NK cell function (Edri et al., 2018). We showed that EBOV-GP also effectively shields ligands to activating NK receptors such as MHC class I polypeptide-related sequence A (MICA) and B7-H6; overall, we showed that EBOV-GP expression by target cells favors the axis reducing NK activation, while not perturbing the NK inhibition axis (Braiman et al., 2006). Following enzymatic and chemical removal of the GP1, we and others showed that GP1 is mediating the shielding effect (Barda-Saad et al., 2005; Braiman et al., 2006). In the current study, we investigated the role of GP1 N-glycosylation on the shielding of ligands to immune receptors, specifically studying the HLA-I and MICA ligands. We demonstrated that the N-glycosylation of both the MLD and GCD domains of GP1 is imperative for the shielding effect and for evading NK cell function. Yet, removal of the GP1 N-glycosylation does not affect the intimate interaction on the cell membrane between GP and its shielded ligand as evident by FRET studies.

## Materials and Methods

### Tissue Culture

HEK293T (ATCC CRL-3216) cells were cultured as recommended by ATCC in DMEMx1 (Gibco, 41965-039) supplemented with 10% fetal calf serum (FCS) (Gibco, 12657-029), 1% l-glutamine (Biological Industries, 03-020-1A), 1% Pen-Strep (BI, 03-031-1B), 1% sodium pyruvate (BI, 03-042-1B), 1% MEM-Eagle (Biological Industries, 01-340-1B), and 1% HEPES 1M (Biological Industries, 03-025-1B). Culture was replaced by newly thawed cells every 3 months and treated every 3 days. To obtain GP and mutated GP-expressing cells, HEK293T cells were plated in 10-cm plates 24 h prior to transfection and transiently transfected using a calcium-phosphate-based reagent with 10 μg DNA per 10-cm plate. Stably transfected HLA-A^*^0201-CFP/NKp46-CFP were cultured using complete DMEM as described above with the addition of 2 μg/ml puromycin (Invivogen, CA, USA).

### Flow Cytometry

Analysis of cell surface marker expression was performed by flow cytometry. HEK293T cells were detached from plastic using non-enzymatic reagent and plated into 96-well u-shaped plates at 10^5^ cells/well. Cells were then stained with 100 μg/ml of the desired mAbs. Viability of cells was determined using 7AAD. HLA-I and MICA were detected with PE-anti human HLA,B,C antibody (BioLogend, 311406) and PE-anti human MICA/MICB antibody (BioLegend, 320906) as the recommended concentration from the company. GP was detected using a biotinylated 3C10 anti GP antibody (Wec et al., 2017) and an Alexa Fluor 647-conjugated streptavidin (Jackson ImmunoResearch, PA, USA). 3C10 is an anti-Ebola Sudan (SUDV) mAb of the IgG2a subclass (Wec et al., 2017). It recognizes a linear epitope in Sudan GP amino acids 351–365 (i.e., within the GP1). Flow cytometry was performed with FACSCanto II (BD Biosciences), and results were analyzed using FlowJo® software (Tree Star).

### GP Mutants Design and Cloning

#### GP Mutants Design

Sequences of E-GP's clustal omega analysis were used to track the N-glycosylation sites; then, mutations were designed by replacing the asparagine amino acid with glutamine in all identified N-glycosylation sites in the GCD or MLD domains or in both domains. Figure S1A details all amino acid sequences in which N-glycosylation site was mutated as well as the codon change. Then, DNA of the GP with the mutated GCD was synthesized and cloned into the expression vector pcDNA3.1, and DNAs of the GP with the mutated MLD, or with the double-mut, were synthesized and cloned into the shuttle vector pUC19 (HyLabs, Rehovot, Israel).

#### GP Mutants Construct Preparation

The insert of the GCD-MLD was digested with *BbvCI* and *EcoNI* from the pUC19 shuttle vectors of the GP-mutated MLD and GP-double-mut. These inserts were ligated into the pcDNA3.1 vector encoding for the GP-mutated GCD that was pre-digested with the same restriction enzymes to remove its GCD-MLD insert. For ligation, vector and insert were mixed (1:7 ratio) and reaction was carried out using T4 kappa rapid ligation enzyme for 10 min at room temperature. Ligation mix was then transformed into DH5α bacterial cells and spread on LB growth plates with ampicillin selection. Five colonies were picked and sent for sequencing.

#### GP WT and Mutants Fused EYFP Preparation

All GP plasmids were digested with *EcoRI* and *SalI* FD (fast digestion) enzymes, as was the vector, pEYFP-N1. The vector/insert was mixed (1:7 ratio) and ligation was carried out using T4 kappa rapid ligation enzyme for 10 min at room temperature. Ligation mix was transformed to DH5α bacterial cells and spread on growth plates with kanamycin selection. Five colonies were picked and sent for sequencing.

#### HLA-A^*^0201 Fused CFP Preparation

pCIpA102-G-HLA-A^*^0201_GFP plasmid was purchased from ADDGENE and amplified with primer + KOZAK FW (29-mer): ggGAATTCgccgccaccatggccgtcatg and primer REV (25-mer): ggGGATCCactcccactttacaagc. It was digested with *EcoRI* and *BamHI* enzymes, as was the PECFP-N1 vector from Clontech. The vector/insert was mixed (1:7 ratio) and ligation was carried out using T4 kappa rapid ligation enzyme for 10 min at room temperature. It was transformed into DH5α bacterial cells and spread on growth plates with kanamycin selection. Five colonies were picked and sent for sequencing.

#### Stable Expression of HLA/NKp46 Fused CFP

HLA2 and NKp46 genes were fused to ECFP reporter gene and cloned into a modified pHAGE2 vector harboring a puromycin selection marker using standard cloning methods. Lentiviruses were created by transient transfection of HEK293T cells using PEI, pHAGE2 vector, and four packaging plasmids, tat, rev, hgpM2, and VSVG, in a ratio of 20:1:1:1:2. Forty-eight hours after transfection, the supernatant was collected and used to infect HEK293Tcells or 3T3NIH by replacing the cell media with LV containing supernatant. Forty-eight hours after infection, cells were selected using 5 μg/ml of puromycin for 4–8 days. After selection, cells were grown in 1 μg/ml of puromycin.

### Microscopy: Co-localization by FRET

The fluorescence resonance energy transfer (FRET) was measured by the donor-sensitized acceptor fluorescence technique as described previously and calculated using custom Matlab scripts (Barda-Saad et al., 2005; Braiman et al., 2006; Jaron-Mendelson et al., 2012). Briefly, the ECFP excitation/EYFP emission, the ECFP excitation/ECFP emission, and the ECFP excitation/EYFP emission images were collected. Calibration curve of background is derived from single- and double-fluorescent protein samples. RETcorr is defined to be the pixel intensity in the corrected FRET image. Then, pixel-by-pixel calculation of FRET is carried out using the equation FRETeff = FRETcorr/(FRETcorr + ECFP) ×100%. Fluorescent images were acquired on a FluoView FV1000 confocal system (Olympus) using a 63 × /1.35 UPLSAPO objective (Olympus).

### CD107a Degranulation Assay for NK Cell Activity

In CD107a degranulation assays, primary NK effector cells (5 × 10^**4**^ cells/well) were co-incubated with target cells (1.5 × 10^5^ cells/well) in the presence of 4 **μ**g/ml APC-conjugated anti-CD107a antibody (BioLegend, 328620) and 30 IU/ml recombinant human IL-2 at 37°C for 5 h. Cells were then stained with 4 **μ**g/ml APC-conjugated anti-CD107a and 4 **μ**g/ml PE-conjugated anti-CD16. Viability of cells was determined using 7AAD by flow cytometry (Canto-II, BD Biosciences), and data were analyzed using FlowJo® software (Tree Star).

### Western Blotting

Expression levels of wild-type and mutant GPs in transfected cells were verified by SDS-PAGE and Western blotting (Figure S1C). Thirty hours post transfection, cells were lysed with ice-cold RIPA lysis buffer [50 mM Tris buffer, pH 8.0, 150 mM NaCl, 1% (v/v) Triton X-100, 0.5% (w/v) sodium deoxycholate, and 0.1% (w/v) SDS] supplemented with protease inhibitors (1.2 mg/ml leupeptin, 1 mM pepstatin A, 100 mM PMSF, and 1 mg/ml aprotinin). The cleared whole-cell lysate was separated by sodium dodecyl sulfate polyacrylamide gel electrophoresis (SDS-PAGE). Proteins were transferred to a 0.2-m nitrocellulose membrane using a semi-dry transfer apparatus (Thermo Fisher Scientific, Power Blotter- Semi-dry Transfer System). Membrane was blocked with Tris-buffered saline containing 0.05% (v/v) Tween-20 (TBST) and 10% (w/v) bovine serum albumin. Membrane was incubated with biotinylated 3C10 anti GP antibody in 4°C overnight, washed with TBST, and then incubated for 1 h at room temperature with HRP-conjugated goat anti-rabbit IgG (#12-348 Sigma-Aldrich). For signal development, membrane washed with TBST before antibody binding was visualized using ECL reagent (#34579, Thermo Fisher Scientific).

### Statistical Analysis

Graphics and statistical analysis were performed using Prism GraphPad 8.0. Statistical analysis of the data was performed using one-way ANOVA (with *p* < 0.05, < 0.01, or < 0.001, as indicated in the figures).

## Results

### N-Glycosylation of MLD and GCD Domains of GP1 Is Involved in the Shielding Phenomenon

**Figure 1:**
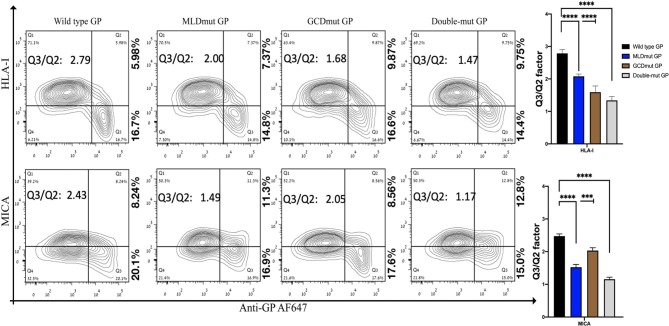
Site-specific mutations, disrupting the N-linked glycosylations in GP1, result in the loss of steric shielding by the EBOV-GP. Flow cytometry analysis shows steric shielding effect over HLA-I (upper panels) and MICA (lower panels) ligands. HEK293T cells were transfected with wild-type GP or with site-specific mutants of GPs with altered N-glycosylation sites (MLD, GCD, or both domains). Thirty hours post transfection, cells (without fixation or permeabilization) were co-stained with mAbs specific for HLA-I or MICA and with mAb specific for EBOV GP (3C10, followed by AF647-labeled secondary). Live cells (7AAD negative) are shown in the contour plots. Q3/Q2 values in the panels are used for evaluating the shielding of MHC-I or MICA by the EBOV GP. End right panels show summary of Q3/Q2 values depicted in the bar chart. Results shown are from one representative analysis of three independent flow cytometry analyses. Graphs representing mean ± SEM. Two-way ANOVA Sidak's multiple comparisons (***P < 0.0005, ****P < 0.0001).

We and others have shown that expression of EBOV-GP shields cell membrane HLA-I from recognition by antibodies or by TCR (Ignatiev et al., 2000; Geisbert et al., 2003; Reed et al., 2004). We then showed that expression of EBOV-GP on target cells also shields ligands to activating NK receptors (Edri et al., 2018). The GP1 fragment of the GP was mediating this shielding since both trypsin and DTT treatments of the cells that remove the GP1 (enzymatically and chemically, respectively) abolished the shielding (Edri et al., 2018). We now investigated whether the N-glycosylation of the GP1 is the major factor mediating this shielding. GP1 is composed of three domains, RBD, MLD, and GCD, in which the MLD and GCD are highly glycosylated, each having several N-glycosylation sites (three and six, respectively, note that Figure S1B shows schematic representation of the GP domains). We mutated all N-glycosylation sites in either the MLD or GCD domains as well as in both domains (MLDmut, GCDmut, and Double-mut, respectively). The size of EBOV-GP and its mutations was then assessed by Western blot (WB) assay; indeed, the three GP mutant types manifested reduced size in the WB that correlated with the number of deleted N-glycosylation sites (Figure S1C). Figure 1 shows the shielding effect of the HLA-I and MICA ligands as compared between wild-type GP and the mutated GPs. The results are shown as a contour plot showing the cell membrane staining of MICA or HLA-I ligands in the *Y*-axis and the cell membrane staining of GP in the *X*-axis. Shielding of ligands by GP is observed when staining of the ligands with ligand-specific Abs is reduced. The experiment is having an internal control since GP transfection is transient and shielding of ligands is observed only for transfected cells stained positively for cell membrane GP (Edri et al., 2018).

All three mutation types reduced the GP-mediated steric shielding, but the Double-mut was the most effective (Figure 1). The observed reduction in steric shielding could not be attributed to reduced expression of the GP mutants on the cell membrane; EBOV-GP and the three mutants showed similar levels of cell surface expression as analyzed by staining with anti-GP antibodies (Figure S2A; overlay of the GP and mutated GP staining). Most of the cells expressing high levels of wild-type GP were found in the Q3 region in the plot reflecting low staining of HLA-I or MICA ligands due to the GP-mediated shielding. For cells expressing high levels of MLDmut GP or GCDmut GP, the cells started drifting toward the Q2 region of the plot, reflecting normal staining of the HLA-I and MICA ligands. The Double-mut GP showed the highest density of cells in the Q2 region. To better evaluate the effect on shielding, we calculate the factor of percent cells in the Q3 section of the contour plot (high GP-positive and ligand-negative for cell membrane staining) divided by percent cells in Q2 section (high GP-positive and ligand positive for cell membrane staining). Higher values of this factor represent a higher shielding effect; i.e., cell membrane stained positively for GP but staining of the MICA and HLA-I ligands on the cell membrane is mostly negative. The Q3/Q2 factor values are listed in the figure panels (Figure 1) and a summary of Q3/Q2 values of three independent experiments is shown in the rightmost panel as a bar histogram (Figure 1). Results, demonstrated that removal of N-glycosylation in either MLD or GCD reduced the shielding effect (lower values of the Q3/Q2 factor). Yet, removal of N-glycosylation in both domains reduced the Q3/Q2 factor by 1.9- to 2-fold as compared to wild-type GP. This was evident for both HLA-I and MICA ligands (Figure 1).

**Figure 2:**
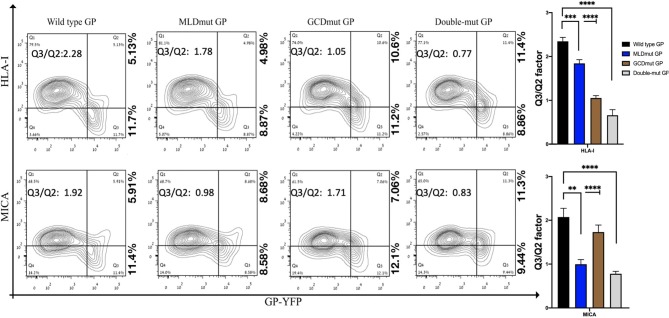
Site-specific mutations, disrupting the N-linked glycosylations in GP1, result in the loss of steric shielding by the EBOV-GP. Flow cytometry analysis shows steric shielding effect over HLA-I (upper panels) and MICA (lower panels) ligands by wild-type and mutated YFP-fused GPs. HEK293T cells were transfected with wild-type GP or with site-specific mutants of GPs with altered N-glycosylation sites (MLD, GCD, or both domains) fused to YFP. Thirty hours post transfection, cells (without fixation or permeabilization) were co-stained with mAbs specific for HLA-I or MICA. Live cells (7AAD negative) are shown in the contour plots. Q3/Q2 values in the panels are used for evaluating the shielding of MHC-I or MICA by the EBOV GP. End right panels show summary of Q3/Q2 values depicted in the bar chart. Results shown are from one representative analysis of three independent flow cytometry analyses. Graphs representing mean ± SEM. Two-way ANOVA Sidak's multiple comparisons (***P < 0.005, ***P < 0.0005, ****P < 0.0001).

The double staining shown in Figure 1 is performed with antibodies recognizing the ligands (HLA-I or MICA) and antibody recognizing the GP1. To compare the effect on shielding between mutations of N-glycosylation sites to a complete removal of the GP1, we had to devise another approach in which antibody to GP1 is not used to monitor the GP expression in the GP-transfected cells. Therefore, we fused either GFP or YFP to the C-terminus of GP; thus, following the trypsin-based enzymatic removal of GP1 from the cell surface, high GP-expressing cells could be monitored due to the GFP or YFP fused to the GP2 domain that persisted in the membrane (and in the cytoplasmic GPpre). In this experimental setup with YFP-fused GPs (wild type or mutated), mutation of the N-glycosylation sites in either MLD or GCD reduced the Q3/Q2 factor by 1.1- to 2.1-fold and mutation of the N-glycosylation sites in both domains reduced the Q3/Q2 factor by 2.3- to 3-fold (Figure 2). This substantial reduction was evident for both the MICA and HLA-I ligands (Figure 2). A summary of Q3/Q2 values of three independent experiments is shown in the rightmost panel as a bar histogram (Figure 2). The somewhat higher reduction in the Q3/Q2 factor observed for N-glycosylation removal in Figure 2 as compared to Figure 1 is an outcome of the different experimental setup, but does not represent a difference in the binding of anti-GP1 mAb between GP and YFP-fused GP. Again, the observed reduction in steric shielding could not be attributed to reduced expression of the FL-conjugated GP mutants on the cell membrane that expressed similarly to the EBOV-GP (Figure S2B; overlay of the GP and mutated GP staining). Moreover, Figure S2C shows that for both wild-type GP, N-glycosylation mutated MLD-GP or GCD-GP or both domains mutated GP, and the binding of the anti-GP1 mAb used in this study (3C10, note section Flow Cytometry) is not affected by the FL fusion or by the mutations in the N-glycosylation sites. The results shown in Figure S2C also nullify the possible scenario that the observed effect of N-glycosylation removal on shielding, as shown in Figure 1, is due to changes in the binding affinity of the anti-GP1 mAb to mutated GPs as compared to wild-type GP.

Trypsin treatment of cells expressing the GFP-fused GP WT removed the GP1 subunit. This treatment reduced the shielding representing Q3/Q2 factor by 1.7, as we previously reported for B7-H6 ligand, which is also shielded by EBOV-GP (Figure 4F in Edri et al., 2018). Thus, since trypsin treatment removes the MLD- and GCD-containing GP1, we can conclude that the N-glycans at the MLD and GCD are the main contributors to the shielding effect. The effect of the trypsin on the level of the cellular target proteins (HLA-I, MICA) is negligible as we previously showed (Edri et al., 2018).

### N-Glycosylation of MLD and GCD Domains of GP1 Is Involved Evasion of NK Function

**Figure 3:**
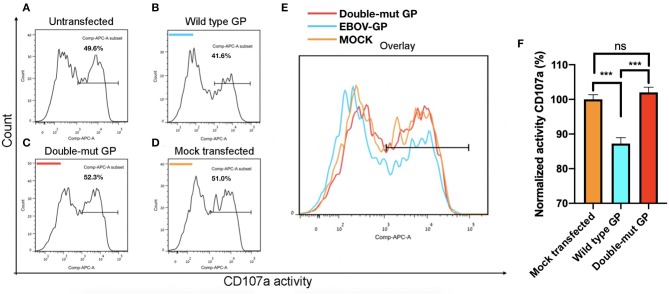
Site-specific mutations, disrupting the N-linked glycosylations in GP, result in the restoration of pNK degranulation. HEK293T cells were either left untransfected **(A)** or transfected with wild-type GP **(B)**, GP glycan deletion mutants **(C)**, or mock vector **(D)**, and cocultured with pNK cells in the presence of 25 U/ml rhIL2 (Edri et al., 2018). Effector cells were sampled and assayed for natural killer cell degranulation marker CD107a. **(E)** Plot shows overlay of CD107a stains of pNK cells cultured with the different target cells. **(F)** Summary plot shows degranulation level of pNK cells cultured with different target cells. Results are from two representative experiments of four replications. Graphs representing mean ± SEM. ***p < 0.001, Ordinary one-way ANOVA multiple comparisons test.

We demonstrated that the GP-mediated steric shielding induces the evasion of NK cell function (Edri et al., 2018). We now assessed whether the N-glycosylation of both the MLD and GCD domains of GP1 is imperative for evading NK cell function. We employed CD107a degranulation assay to assess the function of primary human NK incubated with (i) mock-transfected, (ii) wild type GP-transfected, and (iii) Double-mut GP-transfected HEK293T target cells. The results from the CD107a degranulation assay, shown in Figure 3, demonstrate that wild-type GP-transfected target significantly reduced NK activation as compared to mock-transfected target (Figures 3A–E showing a representative assay and 3F showing a summary of 8 experimental repeats). In contrast to the wild-type GP-transfected target, target cells transfected with Double-mut GP activated NK cells similarly to mock-transfected target cells (Figure 3). Note that transfection levels of the wild-type GP and the Double-mut were very similar (Figure S2); therefore, the differences in NK activation status could not be attributed to the levels of GP expression and should be attributed to the lack of N-glycosylation on GP1 for the Double-mut GP.

### N-Glycosylation of GP1 Is Not Involved in the Co-localization of GP and HLA-A^*^0201 as Assessed by FRET Assay

**Figure 4:**
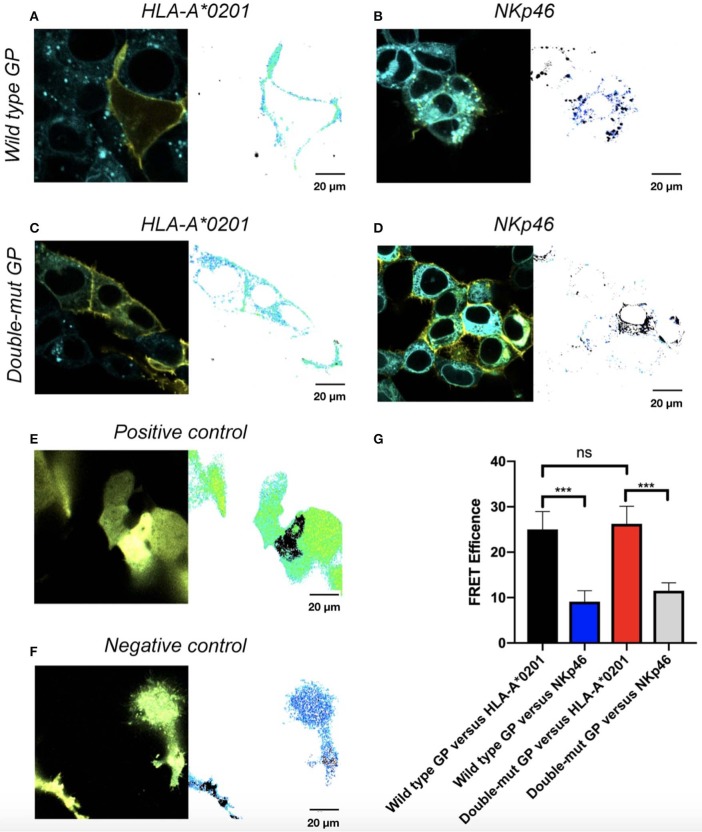
Site-specific mutations, disrupting the N-linked glycosylations in GP, do not affect co-localization of GP with HLA-A*0201. Cells were stably transfected with either CFP-fused HLA-A*0201 **(A,C)** or CFP-fused NKp46 **(B,D)** and then transiently transfected with either YFP-fused wild type GP **(A,B)** or YFP-fused double mutant GP **(C,D)**. Thirty hours post transfection, cells were assayed by confocal microscopy using a 40 × /1.3 UPLSAPO objective (magnification including ocular lens is 400 ×). Summary plot shows FRET efficiency in various samples. Results are representative images from 50 images taken from the experiment. Scale bar (20 μm) is shown at the bottom right corner of each plot. **(E)** Positive control, **(F)** Negative control, **(G)** Summary results. Graphs represent mean ± SEM. ***p < 0.001, ordinary one-way ANOVA multiple comparisons test.

We finally tested whether N-glycosylation of GP1 is involved in the co-localization of GP and its shielded ligands. We employed the FRET assay that is generally used to determine interactions between proteins, by using two specific pairs of fluorescence proteins, in our case CFP-donor and YFP-acceptor. FRET was used to determine the distance between wild-type GP and its shielded HLA-I and then compared to the distance between Double-mut GP and the same HLA-I. Both GP and the HLA-I allele tested were fused to YFP (wild type GP or Double-mut-GP) and CFP (HLA-A^*^0201). We then stably transfected HEK293T with the CFP-HLA-A^*^0201 as well as with a CFP-conjugated control protein that is not shielded by wild-type GP (CFP-NKp46). These stable transfections were carried out to avoid different transfection efficiencies that can skew the results if we would have taken the approach of double transfection of GP and HLA-A^*^0201. The stable transfectants (CFP-HLA-A^*^0201 or CFP-control protein) were then transfected with YFP-GPs. As predicted, a high FRET efficiency was measured for wild-type GP and HLA-A^*^0201, as compared to wild-type GP and control protein not shielded by GP (Figures 4A,B). For each panel, the image on the left-hand side shows the overlay of the two proteins and the image on the right side illustrates the calculation of FRET efficiency, with yellow indicating high, blue indicating low, and black showing saturation of any fluorescence protein. The pictures for wild-type GP and HLA-A^*^0201 depict very high FRET efficiency in the membrane of the cell, indicating that the two proteins are very close (1–10 nm) in the membrane of the cells (Figure 4A). When CFP-Double-mut GP was employed, similar results were obtained. The pictures for Double-mut GP and HLA-A^*^0201 depict very high FRET efficiency in the membrane of the cell (Figure 4C), while no FRET was observed for the Double-mut GP and the control CFP-protein (Figure 4D). Figures 4E,F show the FRET-positive and -negative controls, respectively. Positive control represents transfection with a plasmid encoding for YFP-CFP-fused protein and negative control represents random FRET intensity as transfection is performed with both CFP-encoding and YFP-encoding plasmids. Figure 4G shows the summary of near 50 FRET pictures for each pair; no significant difference is observed between FRET efficiency of wild-type GP and HLA-A^*^0201 as compared to Double-mut GP and HLA-A^*^0201. No interactions are observed between both GP types and the control non-shielded protein.

## Discussion

Ebola virus is exceptionally lethal, in part due to immune-evasion mechanisms (Lori et al., 2015). The steric shielding of HLA-I and MICA ligands by EBOV-GP employed as a viral escape mechanism had been reported (Takada et al., 2000; Francica et al., 2010; Edri et al., 2018). Viral evasion strategy that sterically interferes with immune receptors is a broad phenomenon found in various families of viruses (Jones et al., 1996; Wiertz et al., 1996a,b; Ahn et al., 1997; Hengel et al., 1997; Gruhler et al., 2000; Gewurz et al., 2001; Li et al., 2010; Goulder and Walker, 2012; Mann et al., 2013; Ressing et al., 2015). The HLA-I and the MICA are ligands that constitute major targets in viral-mediated evasion of immunity. Nonetheless, their modulation must take into account the opposing role some ligands play in T cell vs. NK cell activation. A distinct example to this is the case of HLA modulation by viruses. While down-modulation of HLA may grant virally infected cells immunity against T cell activity, it may also expose it to NK activity and vice versa (Brusilovsky et al., 2012). T/NK cover-up may enforce HLA-I-based viral evasion mechanisms, coupling with a complementary mechanism to address the consequent activation of either NK or CTL cells. Prominent examples of viruses modulating HLA-I are those of the flaviviruses and cytomegalovirus (Jones et al., 1996; Wiertz et al., 1996a,b; Ahn et al., 1997; Hengel et al., 1997; Gruhler et al., 2000; Gewurz et al., 2001; Li et al., 2010; Goulder and Walker, 2012; Mann et al., 2013; Ressing et al., 2015). Importantly, in the case of Flaviviruses, HLA-I is upregulated early after infection, hinders NK cells, and allows for acute viremia well before the adaptive response may be established. Such an early stage viremia is a crucial condition for the viral infection of its arthropod vector (Hershkovitz et al., 2008). Moreover, the long-term survivors were found to mount effective antibodies specifically against the viral GP (Radinsky et al., 2017), and this was also shown in a mouse model (Ren et al., 2018). EBOV GPs were also reported recently to have additional anti-T cell activities by directly binding CD4+ T cell through interaction of GP with TLR4, resulting in significant upregulation of pathways associated with interferon signaling that leads to T cell apoptosis or necrosis (Iampietro et al., 2017).

Alazard-Dany et al. (2006) published in 2006 that upon transient transfection of Kunjin replicon GP-expressing cells with GP-coding plasmid DNA, GP was overexpressed; this overexpression induced “downregulation” of surface molecules and demonstrated that downregulation of the surface markers are the late events in EBOV infection (Alazard-Dany et al., 2006). Others and us have shown that this reported “downregulation” of surface molecules is in fact a steric shielding mediated by the GP (Colonna and Samaridis, 1995; Francica et al., 2010; Noyori et al., 2013). Thus, steric shielding by Ebola GP was demonstrated in virally infected cells and not only in GP-transfected cells and steric shielding phenotype in infected cells shows a great resemblance to that observed in GP-transfected cells (Alazard-Dany et al., 2006). In this study, we show that the steric shielding is mediated by the N-glycans conjugated to the MLD and GCD domains. Noyori et al. proposed steric shielding efficiency of different Ebola virus species and their relative pathogenicity (Noyori et al., 2013). Yet, the number of N-glycans among GPs of various Ebola virus species is similar, while pathogenicity varies. Indeed, the same authors have suggested that while the GP high-glycosylation phenotype is a necessary condition for viral pathogenicity, additional parameters (like flexibility and stability) of the GP are involved in the differential pathogenicity of the various species (Noyori et al., 2013). Groseth et al. proposed another explanation, demonstrating that GP alone is not sufficient to confer a lethal phenotype and that other factors are needed for the acquisition *in vivo* of full virulence (Groseth et al., 2012).

Cryo-EM analysis of Ebola GP reveals that the MLD is occluding the glycan cap and the receptor-binding region (Beniac and Booth, 2017). To expose the receptor binding region of GP, the need for removal of both MLD and GCD domains is suggested. In addition, we observed that removal of the N-glycans did not affect the GP binding of anti-GP monoclonal antibodies; others have shown the same (Francica et al., 2010). Therefore, it is possible that removal of the N-glycans of either MLD or GCD or both domains did not alter the core structure of the GP and that this core is not involved in the steric shielding but rather in the binding to its ligand as suggested (Beniac and Booth, 2017).

As aforementioned, we further decipher the mechanism involved in steric shielding by EBOV-GP. Others and us showed that the GP1 part of the EBOV GP is responsible for the steric shielding of HLA-I (Francica et al., 2010; Edri et al., 2018). We then showed that GP1 also shields ligands to activating NK receptors (Edri et al., 2018). GP1 is highly N-glycosylated (Lennemann et al., 2014). We and others have shown that N-glycosylation on NK receptors is involved in the binding of NK receptors to viral GPs (Cerwenka and Lanier, 2001; Zilka et al., 2005; Higai et al., 2011; Rosental et al., 2011; Brusilovsky et al., 2012, 2013; Higai and Matsumoto, 2012; Matta et al., 2013). Yet, in this study, we showed that it is the N-glycosylation on the EBOV-GP that induces viral evasion through mediating shielding of ligands to NK receptors. Involvement of N-glycans of viral GP in immune evasion was shown for several viral families yet in different mechanisms. Hepatitis C virus (HCV) envelope GP heterodimer, E1E2, is highly N-glycosylated, and one of the key functions of these N-glycans is to sterically shield antigenic epitopes on the E1E2 (Lavie et al., 2018). Similarly, the Porcine Reproductive and Respiratory Syndrome virus (PRRSV) has a complex design of four GPs flanked with 15 N-glycans in their ectodomain that are also involved in shielding antigenic epitopes of the viral GPs (Stoian and Rowland, 2019). Shielding of GP antigenic epitopes by its N-glycans was also reported for the envelope proteins of hepatitis B virus and for the N-glycans of influenza virus hemagglutinin (Julithe et al., 2014; Pentiah et al., 2014). A different mechanism for immune evasion mediated by N-glycans of the viral envelop protein was suggested for the Zika virus in which the N-glycans are neutralizing the reactive oxygen species pathway of the mosquito immune system.

Our results demonstrate the imperative contribution of N-glycans of the EBOV-GP1 to immune evasion through mediating the steric shielding of ligands to immune receptors expressed on the cell membrane in which the GP is also expressed. N-glycans from both the MLD and GCD domains of the GP1 were involved in this shielding. Mutation of the GP1 N-glycosylation sites was sufficient to abolish the interference to NK function mediated by wild-type GP. Interestingly, mutation of the GP1 N-glycosylation sites did not have any effect on the intimate interaction of EBOV-GP and MHC-I ligands; no difference in FRET efficiency was detected between wild-type GP and the Double-mut GP when the distance between GP and HLA-I was assessed. It could be that the interaction between GP and HLA-I is established through binding of GP2 and HLA-I either in the transmembrane or in the cytoplasmic domains of the GP2. To summarize, we characterized the involvement of GP1 N-glycans in the steric shielding of cell membrane ligands to immune receptors by the EBOV-GP and showed their important contribution to this phenomenon and to EBOV GP-mediated immune evasion.

## Data Availability Statement

The datasets analyzed in this article are not publicly available. Requests to access the datasets should be directed to iraqi@post.bgu.ac.il.

## Author Contributions

MI, AE, LL, and AP initiated the project. MI, AE, AB, KK, and AP planned, performed, and analyzed experiments. MI, AE, YG, PB, AC, AO, AB, and AP designed and prepared required cell cultures, expression vectors, and reagents. MI, AE, RG, and AP wrote the manuscript.

### Conflict of Interest

The authors declare that the research was conducted in the absence of any commercial or financial relationships that could be construed as a potential conflict of interest.

## Supplementary Material

Figure S1 Schematic illustration of GP Glycosylation sites and design of mutations. **(A)** Illustration showing the design of mutants. Codon alteration is noted over the amino acid sequence. N-Glycosylation sites are highlighted in yellow and altered amino acids are highlighted in red. **(B)** Glycosylation sites of the MLD and GCD domains; N-Glycosylation sites are marked with the “Y” mark. **(C)** WB analysis of HEK293T cells; cells were transfected with either WT GP or GP mutants. Glycosylation loss caused change in migration pattern.

Figure S2 **(A)** Overlay of WT/mutant GP transfected HEK293T cells stained with biotinylated anti GP Ab and streptavidin AF647. **(B)** Overlay of WT/mutant YFP fused GP transfected HEK293T cells. **(C)** Linear correlation between YFP-fused wild type GP and glycan deletion mutants of GP. HEK293T cells were transfected with wild type GP or with glycan deletion mutants of GP created by site directed removal of N-Glycosylation sites (MLD, GCD or both domains). Thirty hours post transfection, cells (without fixation or Permeabilization) were stained with a biotinylated 3C10 anti GP antibody (Edri et al., 2018), followed by allophycocyanin-conjugated streptavidin. Panels show the YFP-conjugated glycan deletion mutants of GP and the YFP-conjugated wild type GP.
