# Supplementary figures and images for: N-Glycans Mediate the Ebola Virus-GP1 Shielding of Ligands to Immune Receptors and Immune Evasion
Source: Front Cell Infect Microbiol. 2020 Mar 6;10:48. doi: 10.3389/fcimb.2020.00048 (PMC7068452; doi:10.3389/fcimb.2020.00048)

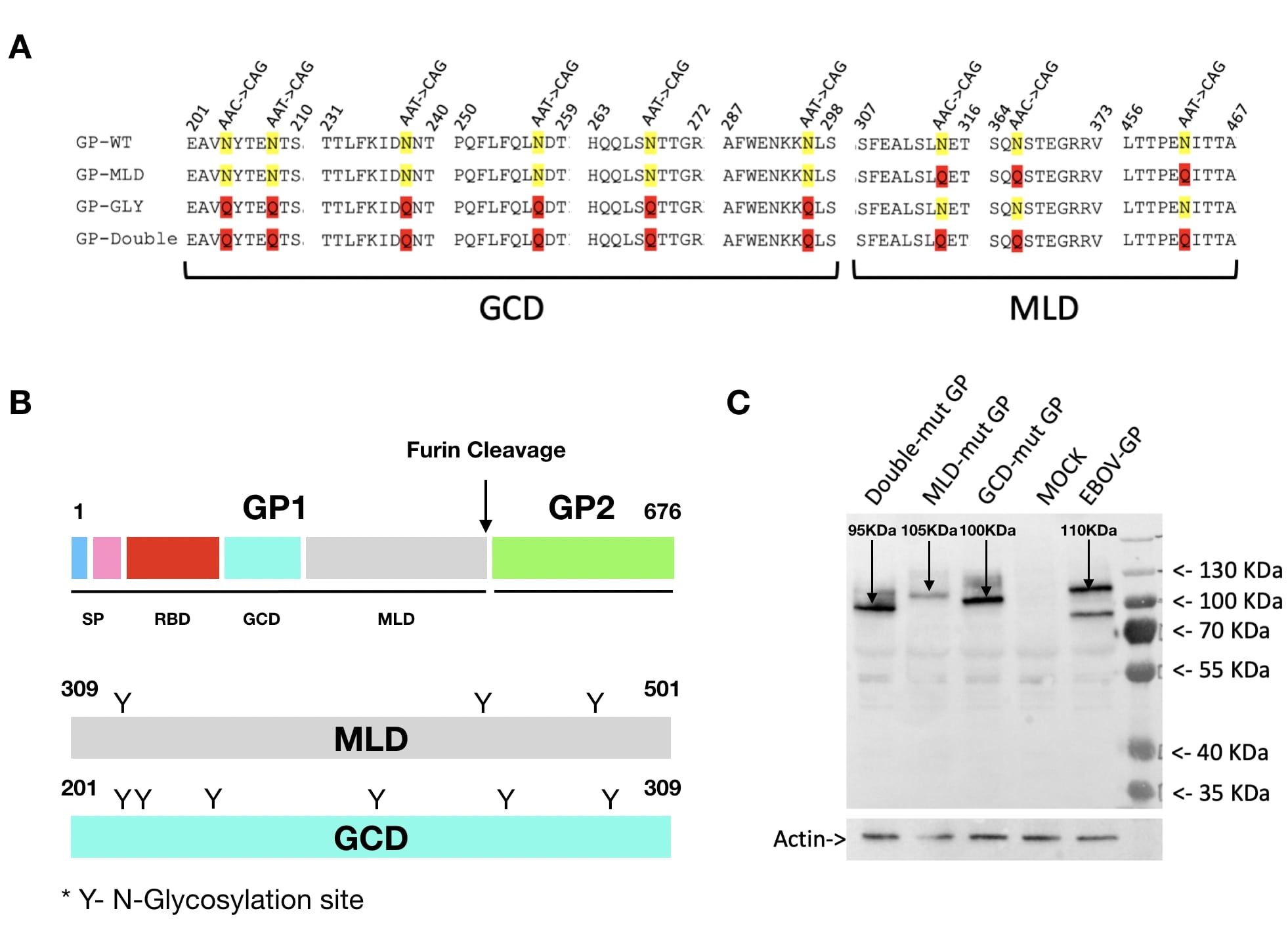

Supplement: Figure S1 — Schematic illustration of GP Glycosylation sites and design of mutations. (A) Illustration showing the design of mutants. Codon alteration is noted over the amino acid sequence. N-Glycosylation sites are highlighted in yellow and altered amino acids are highlighted in red. (B) Glycosylation sites of the MLD and GCD domains; N-Glycosylation sites are marked with the “Y” mark. (C) WB analysis of HEK293T cells; cells were transfected with either WT GP or GP mutants. Glycosylation loss caused change in migration pattern. [file Image_1.JPEG]

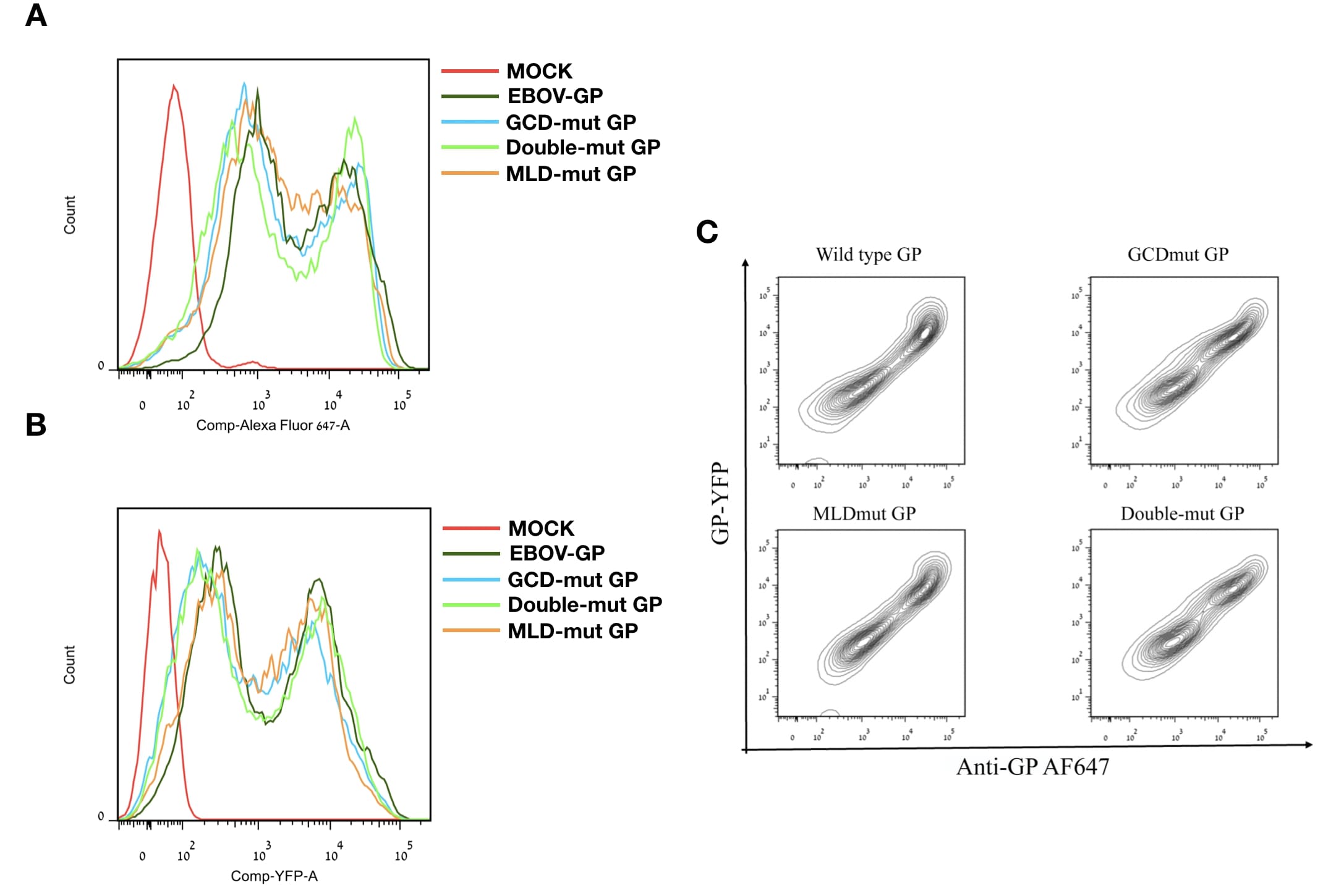

Supplement: Figure S2 — (A) Overlay of WT/mutant GP transfected HEK293T cells stained with biotinylated anti GP Ab and streptavidin AF647. (B) Overlay of WT/mutant YFP fused GP transfected HEK293T cells. (C) Linear correlation between YFP-fused wild type GP and glycan deletion mutants of GP. HEK293T cells were transfected with wild type GP or with glycan deletion mutants of GP created by site directed removal of N-Glycosylation sites (MLD, GCD or both domains). Thirty hours post transfection, cells (without fixation or Permeabilization) were stained with a biotinylated 3C10 anti GP antibody (Edri et al., 2018), followed by allophycocyanin-conjugated streptavidin. Panels show the YFP-conjugated glycan deletion mutants of GP and the YFP-conjugated wild type GP. [file Image_2.JPEG]
